# Correction: Long non-coding RNA PVT1 promotes tumor progression by regulating the miR-143/HK2 axis in gallbladder cancer

**DOI:** 10.1186/s12943-024-01935-x

**Published:** 2024-01-15

**Authors:** Jianan Chen, Yan Yu, Hua Li, Qiuyue Hu, Xiaolong Chen, Yuting He, Chen Xue, Fang Ren, Zhigang Ren, Juan Li, Liwen Liu, Zhenfeng Duan, Guangying Cui, Ranran Sun

**Affiliations:** 1 Precision Medicine Center, The First Affiliated Hospital of Zhengzhou, University, Zhengzhou, 450052 China; 2 Key Laboratory of Clinical Medicine, The First Affiliated Hospital of Zhengzhou University, Zhengzhou, 450052 China; 3 Sarcoma Biology Laboratory, Department of Orthopaedic Surgery, David Geffen School of Medicine at University of California Los Angeles, Los Angeles, CA 90095 USA; 4 National Engineering Laboratory for Internet Medical System and Application, The First Affiliated Hospital of Zhengzhou University, Zhengzhou, 450052 Henan China

**Correction: Mol Cancer 18, 33 (2019)**

**https://doi.org/10.1186/s12943-019-0947-9**

Following publication of the original article [1], the authors, after thorough checking the original data, they have found three unintentional duplication in this paper, the authors requested to update the figures as stated below.

- We request to replace the misused image in Fig. 2K with the correct image

**Fig. 2:**
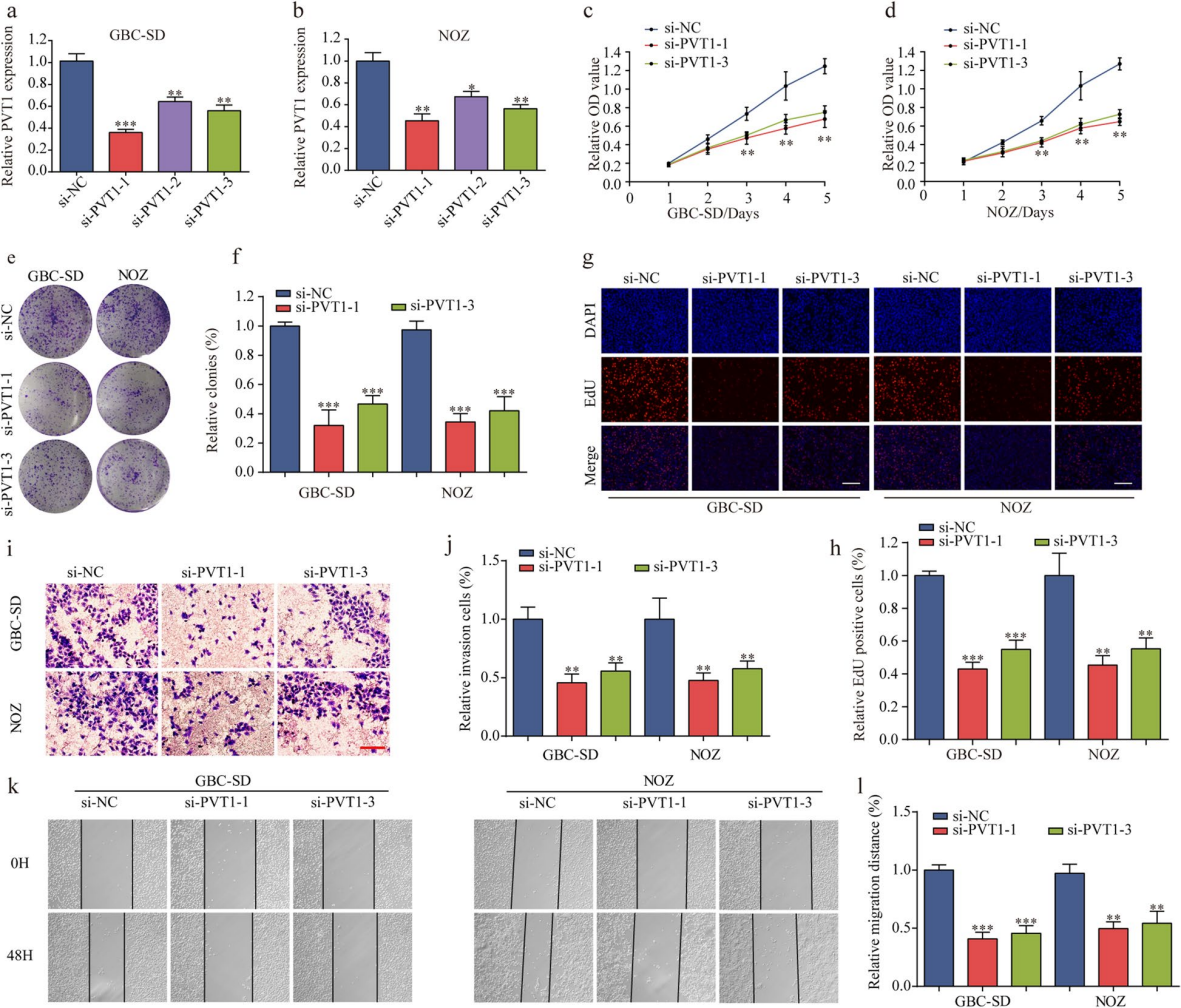
The effect of PVT1 on GBC cells biological behavior in vitro. **a-b** PVT1 expression was knocked down by three siRNAs targeting PVT1 in GBC-SD and NOZ cells. **c-d** Knockdown of PVT1 significantly decreased cell proliferation compared with si-NC cells using CCK-8 assay. **e–f** Colony numbers of GBC-SD and NOZ cells transfected with si-PVT1 were significantly lower than in those transfected with si-NC. **g-h** EdU assay showed that suppression of PVT1 attenuated the proliferation of GBC-SD and NOZ cells (magnification, × 100). Scale bar, 100 μm. **i-l** PVT1 suppression impaired GBC cell invasion and migration, as measured by a transwell assay (magnification, × 100). Scale bar, 100 μm and wound healing assay, respectively. *P < 0.05, **P < 0.01, ***P < 0.001. Error bars indicate mean ± SD

 

- We request to replace the misused images in Fig. 5d and 5i with the correct images

**Fig. 5:**
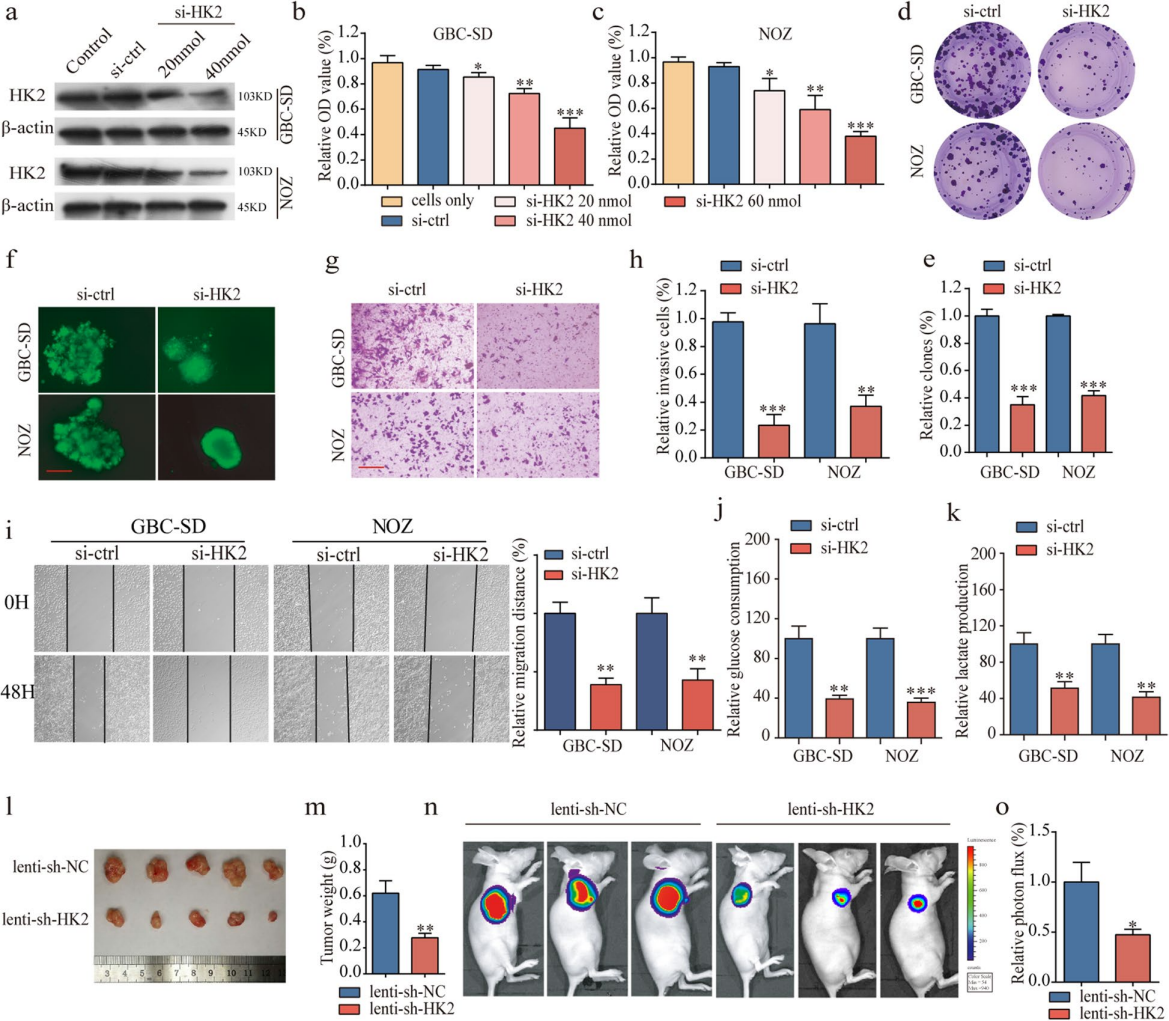
HK2 promotes cell proliferation, invasion and migration in vitro and tumor growth in vivo. **a** The protein levels of HK2 in GBC cells after knockdown by si-HK2. **b-e** Knockdown of HK2 significantly decreased cell proliferation compared with si-ctrl cells by proliferation assays. **f** Formation of spheres from GBC cells transfected with si-HK2 accessed by three-dimensional cell culture (magnification, × 200, scale bar, 50 μm). **g-i** HK2 suppression impaired GBC cell invasion and migration, as measured by transwell assay (magnification, × 100). Scale bar, 100 μm and wound healing assays. **j-k** Glucose consumption and lactate production were significantly decreased after HK2 knockdown. **l-m** Tumor volume and weight in the lenti-sh-HK2 group were significantly lower than those in the lenti-sh-NC group. **n** Images of tumor formation were performed by a live imaging system detecting the luciferase signal. **o** The luciferase activity in the lenti-sh-HK2 group was lower than that in the lenti-sh-NC group. *P < 0.05, **P < 0.01, ***P < 0.001. Error bars indicate mean ± SD

- We request to replace the misused image in Fig. 8e with the correct images

**Fig. 8:**
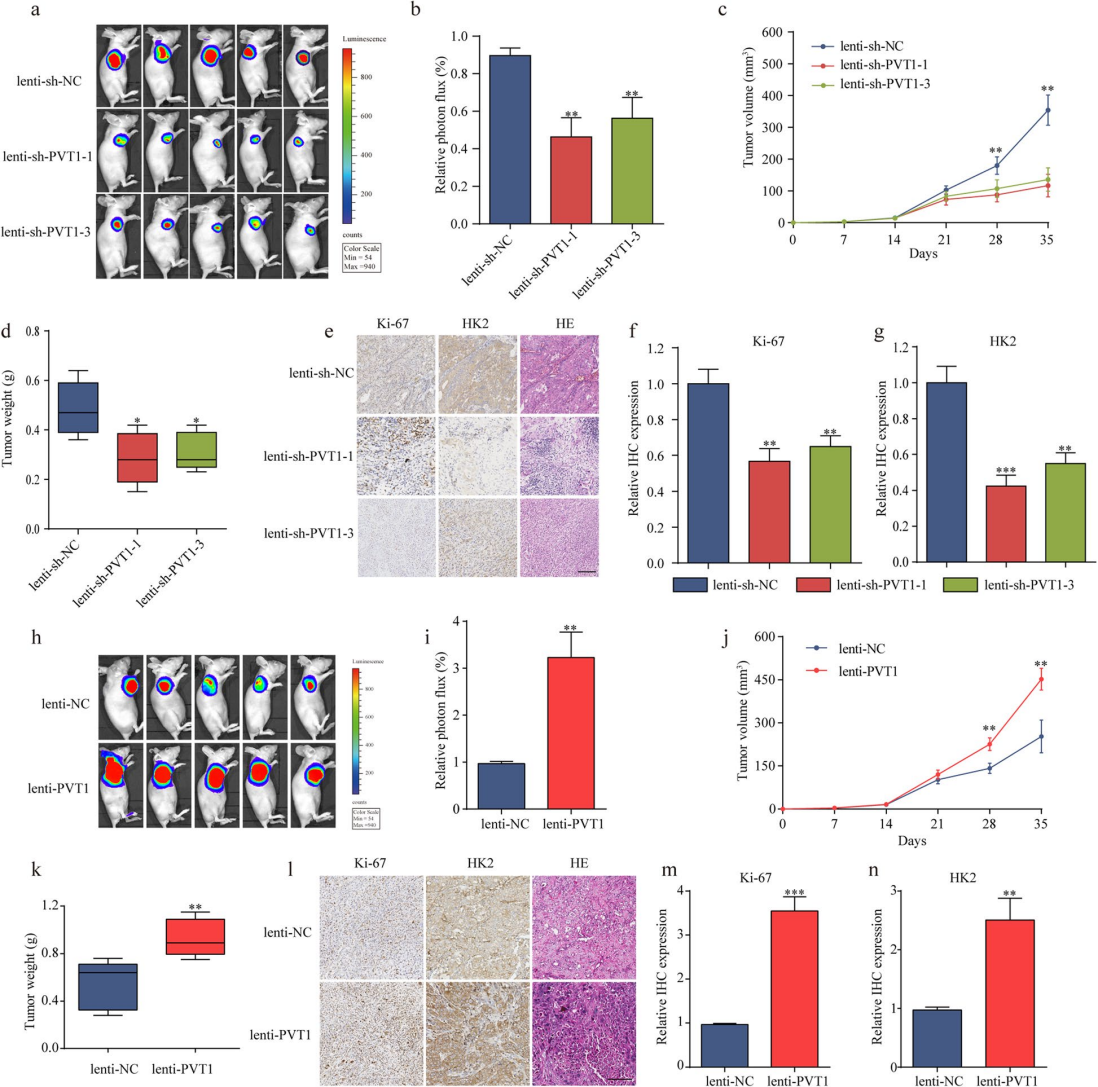
PVT1 promotes tumor growth in vivo. **a** Images of tumor formation were performed by a live imaging system detecting the luciferase signal. **b** The luciferase activity in the lenti-sh-PVT1 group was lower than in the lenti-sh-NC group. **c-d** The volumes and weight of lenti-shPVT1 cell-derived xenograft tumors were markedly lower than those of the lenti-sh-NC group. **e–g** Sections of xenograft tumors stained with hematoxylin and eosin (H&E) as well as immunohistochemical staining for HK2 and Ki-67 (magnification, × 200). Scale bar, 100 μm. **h** Representative images of tumor formation of the lenti-NC group and lenti-PVT1 group. **i** The luciferase activity in the lenti-PVT1 group was higher than that in the lenti-NC group. **j-k** The volumes and weight of lenti-PVT1 cell-derived xenograft tumors were markedly higher than those of the lenti-NC group. **l-n** The levels of Ki67 and HK2 were much higher after PVT1 overexpression (magnification, × 200). Scale bar, 100 μm. *P < 0.05, **P < 0.01, ***P < 0.001. Error bars indicate mean ± SD

To ensure the reliability of the experimental conclusion, three repeated experiments were performed again by different authors from their team which has no conflict of interest. The correction does not change the results and scientific conclusions of this article. We sincerely apologize to the editor, reviewers and readers for the errors and any confusion it may have caused. We want to make a correction to this error as soon as possible.
